# Robust mode-locking in all-fiber ultrafast laser by nanocavity of two-dimensional heterostructure

**DOI:** 10.1038/s41377-025-02018-2

**Published:** 2025-09-03

**Authors:** Jiahui Shao, Guangjie Yao, Xuecheng Wu, Kaifeng Lin, Shaoyi Zhang, Xu Cheng, Ding Zhong, Chang Liu, Can Liu, Fengqiu Wang, Kaihui Liu, Hao Hong

**Affiliations:** 1 State Key Lab for Mesoscopic Physics and Frontiers Science Center for Nano-optoelectronics, School of Physics, Peking University, 100871 Beijing, China; 2 Academy for Advanced Interdisciplinary Studies, Peking University, 100871 Beijing, China; 3 School of Electronic Science and Engineering, Nanjing University, 210023 Nanjing, Jiangsu China; 4 Group for Fibre Optics, École Polytechnique Fédérale de Lausanne, 1015 Lausanne, Switzerland; 5 Key Laboratory of Quantum State Construction and Manipulation (Ministry of Education), School of Physics, Renmin University of China, 100872 Beijing, China; 6 International Centre for Quantum Materials, Collaborative Innovation Centre of Quantum Matter, Peking University, 100871 Beijing, China; 7 Songshan Lake Materials Lab, 523808 Dongguan, Guangdong China; 8 Interdisciplinary Institute of Light-Element Quantum Materials and Research Centre for Light-Element Advanced Materials, Peking University, 100871 Beijing, China

**Keywords:** Fibre lasers, Mode-locked lasers, Optical properties and devices

## Abstract

The fiber-based saturable absorber (SA) that enables mode-locking within a ring cavity serves as the core component of the ultrafast all-fiber lasers. However, the integration of SAs onto fibers with high compactness suffers from imbalanced saturable absorption properties and unstable mode-locking performance. Here, we present a robust mode-locking SA by integrating a nanocavity composed of a two-dimensional graphene heterostructure on the fiber end facet. We demonstrate a significant reduction in the saturation intensity (~65%) and improved soliton dynamic processes through precise modulation of the optical field within the heterostructure. The designed heterostructure facilitates the formation of a stable single-soliton state for robust mode-locking. A high tolerance to intracavity polarization variations is achieved in the heterostructure-SA (~85% compared to 20% for bare graphene). Our designed heterostructure-SA represents an important advancement in the development of ultracompact mode-locked all-fiber lasers, offering enhanced integrability and stability.

## Introduction

Mode-locked fiber lasers with ultrafast temporal information, ultrahigh peak energy, and exceptional stability have emerged as pivotal devices in modern optical research and industrial applications, including ultrafast probing, micromachining, and telecommunications^1,2^. The heart of ultrafast lasers lies in mode-locking components, which are responsible for coupling and phase-locking the cavity modes to convert continuous-wave light into pulsed laser output. Among the diverse mode-locking components available, real saturable absorbers (SAs) are widely utilized because of their unique advantages of self-starting operation, robust and maintenance-free characteristics^3–5^. Although various material systems—including organic dyes^6^, color filter glasses^7^, ion-doped crystals^8^, and semiconductors^9^—can function as SAs, achieving a high-quality mode-locking state remains challenging in most cases. This issue is due primarily to the intricate balance required among key parameters such as the modulation depth (*α*_0_), saturation intensity (*I*_s_), nonsaturable absorption (*α*_ns_), and damage threshold (*P*_th_)^10^. Consequently, semiconductor saturable absorption mirrors, in which III–V semiconductor multiple quantum wells grown on distributed Bragg reflectors are employed to control saturable absorption, have become the most commercially favored SAs in fiber lasers. However, semiconductor saturable absorption mirrors typically require free-space alignment for coupling, which compromises the all-fiber structure^11^. To realize a compact and fully all-fiber-integrated design, it is imperative to explore both new materials and novel architectures for directly integrating SAs onto optical fibers.

The advent of low-dimensional materials and advancements in nanofabrication techniques have opened exciting possibilities for developing fiber-based SAs^12–15^. Among these materials, graphene stands out as particularly promising. Its ultrafast recovery time (<100 fs) and linear energy dispersion enable mode-locking across a broad spectral range (from visible to infrared) with ultrashort pulses (<90 fs) and high repetition rates (>9 GHz)^16–19^. The atomic-level flatness of graphene guarantees perfect van der Waals integration with optical fibers without disturbing the optical mode. Previously, different coupling strategies, including transmission coupling and evanescent-wave coupling, have been developed for graphene-SA integration^20–24^. Various manipulation methods and configurations—such as polarization control^25^, heterostructures^26^, gating^27^, and interference effect^28^—have been explored. However, diverse output states have been reported from graphene-SA^29,30^, including continuous-wave emission, single-pulse mode-locking, harmonic mode-locking, and pulse splitting, complicating the overall scenario for practical utilization. Thus, a comprehensive and unified understanding of the mechanisms governing soliton formation with graphene-SA is crucial. Such insights will be instrumental in guiding the future optimization and advancement of graphene-based SAs.

Here, we provide detailed real-time information on the self-starting process and inherent internal evolution of solitons in a fiber laser mode-locked by graphene. The energy competition between multiple background pulses increases the possibility of multi-soliton formation. To improve the soliton dynamics, we design an optical fiber-integrated heterostructure composed of MoS_2_-BN-graphene-BN-MoS_2_ to balance the saturable absorption properties. Through precise modulation of the optical field, the designed heterostructure can effectively suppress the nonsoliton components near the central wavelength and mitigate multiple competing background pulses. Therefore, a robust single-soliton mode-locking all-fiber laser is successfully obtained.

## Results

### Design of a two-dimensional (2D) heterostructure saturable absorber

**Fig. 1:**
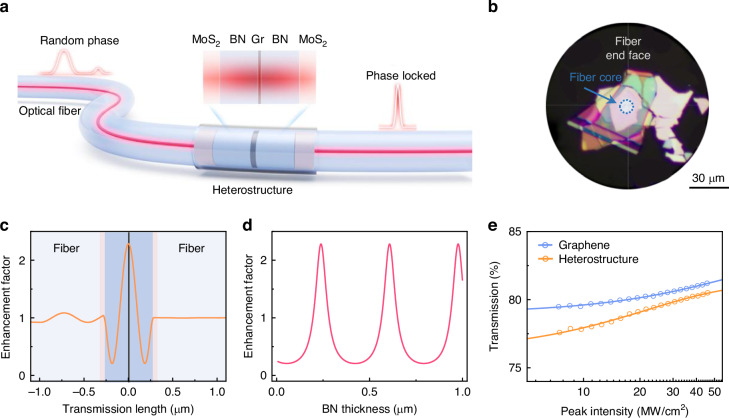
Fiber-integrated heterostructure-SA. **a** Schematic representation of the MoS_2_-BN-graphene-BN-MoS_2_ heterostructure embedded between optical fiber end facets. The heterostructure forms a nanocavity with a nonuniform optical field distribution. The input laser with random phases is modulated into phase-locked pulses with the interaction of the heterostructure-SA. **b** Optical image of the end facet of an optical fiber with the heterostructure (with an BN thickness of 240 nm) integrated. **c** Internal optical field intensity distribution of the heterostructure. The optical field intensity in graphene can be enhanced by ~230% with an BN thickness of 240 nm. **d** Optical field intensity enhancement factor (*I*_*cavity*_/*I*_graphene_) as a function of BN thickness, simulated with COMSOL. **e** Transmission measurements (circles) of graphene (blue) and the heterostructure (with an BN thickness of 240 nm, orange) as a function of the pump peak intensity. The solid curves represent the fitted results, with a modulation depth (α_0_) of 4.2% and a saturation intensity (*I*_s_) of 62.9 MW/cm^2^ for bare graphene and values of 5.0% and 22.0 MW/cm^2^ for the heterostructure

In our experiments, 2D materials were mechanically exfoliated and sequentially stacked to form MoS_2_-BN-graphene-BN-MoS_2_ heterostructures. These heterostructures were then transferred onto the end facet of a single-mode optical fiber and aligned with another fiber (Fig. 1a, b and Figs. S1, S2; see “Materials and methods” section for fabrication details). Owing to the disparity in the refractive indices of MoS_2_ and BN (*n*MoS_2_ = 4.1 and *n*_BN_ = 2.1 at 1550 nm), the heterostructure forms a nanocavity with a nonuniform optical field distribution (Fig. 1c). Our simulations indicate that the optical field intensity in the graphene layers can be either suppressed or enhanced by varying the thickness of the BN layers (Fig. 1d). The enhancement factor, defined as *I*_cavity_/*I*_graphene_, varies from 20% (with an BN thickness of 55 nm) to 230% (with an BN thickness of 240 nm). Fiber-integrated SA components with bare graphene and heterostructures were experimentally prepared. The corresponding nonlinear absorption curves are presented in Fig. 1e. As the pump peak intensity increases, their total transmission monotonically rises and can be modeled by the following equation:

1 $$T\left(I\right)=1-{\alpha }_{0}\cdot \exp \left(\frac{-I}{{I}_{S}}\right)-{\alpha }_{{\rm{ns}}}$$

where *I* represents the pump peak intensity, *α*_0_ is the modulation depth, *α*_ns_ is the nonsaturable absorption, and *I*_s_ is the saturation intensity.

From the fitting, we find a modulation depth of 4.2% and a saturation intensity of 62.9 MW/cm^2^ for the SA component with bare graphene. Because of the enhanced optical field, the SA component with the heterostructure with an BN thickness of 240 nm has a modulation depth of 5.0% and a saturation intensity of only 22.0 MW/cm^2^. In contrast, no evident saturable absorption effect is observed for the SA component with the heterostructure with a suppressed optical field intensity (with an BN thickness of 55 nm, Fig. S3).

### Performance of graphene-based SA mode-locked fiber lasers

**Fig. 2:**
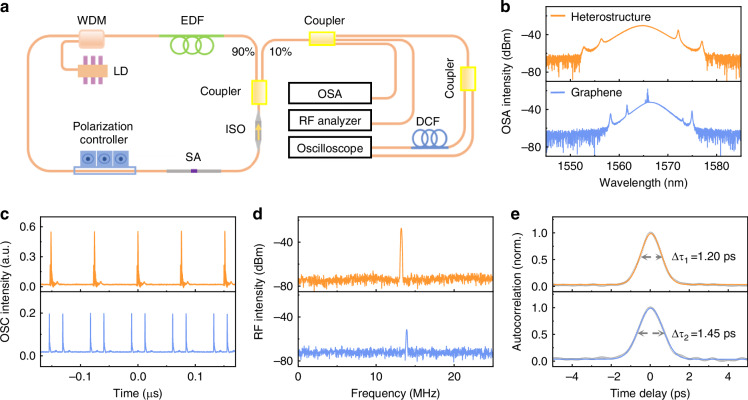
Ultrafast all-fiber lasers based on the bare graphene-SA and heterostructure-SA **a** Schematic of the mode-locked all-fiber laser and measurement system. The optical components include a laser diode (LD), a wavelength-division multiplexer (WDM), a dispersion compensation fiber (DCF), an erbium-doped fiber (EDF), an isolator (ISO), a saturable absorber (SA), and an optical spectrum analyzer (OSA). **b** Spectra of the output lasers with the bare graphene-SA and heterostructure-SA. There is an obvious nonsoliton component near the central wavelength in the spectrum of the graphene-SA, in contrast to that of the heterostructure-SA. **c** Output pulse trains from the all-fiber lasers. The repetition rate is 13.2 MHz for the heterostructure-SA and 14.0 MHz for the graphene-SA. The pulse train in graphene is accompanied by pulse splitting. **d** RF spectra measured. The signal-to-noise ratios of the heterostructure-SA and graphene-SA are 45 dB and 22 dB, respectively. **e** Autocorrelation traces with FWHM of ~1.20 ps for the heterostructure-SA and ~1.45 ps for the graphene-SA, fitted by Gaussian functions

We subsequently designed an all-fiber ring cavity and integrated the bare graphene and heterostructure for mode-locking, respectively (Fig. 2a). The output laser spectra from bare graphene and the heterostructure have similar central wavelengths and spectral bandwidths at a pump power of 62 mW (Fig. 2b). The spectra display clear Kelly sidebands, indicating conventional soliton generation. In the bare graphene-SA spectrum, there are obvious nonsoliton components near the central wavelength at 1565.8 nm. This component significantly influences the soliton behavior through long-range soliton interactions^31^, eventually leading to pulse splitting, as evidenced by the blue trace in Fig. 2c. For the heterostructure system, the spectrum broadening (Fig. 2b) facilitates the generation of shorter pulse duration. Simultaneously, the suppression of the nonsoliton components enhances the stability of the single-soliton mode-locked output (Fig. 2c). In addition, the radio frequency (RF) spectrum reveals a signal-to-noise ratio of 45 dB for the heterostructure (at 13.2 MHz), demonstrating a significant improvement over the value of 22 dB for bare graphene (at 14.0 MHz, Fig. 2d). For more precise temporal characterization, Fig. 2e presents autocorrelation traces of mode-locked pulses from the heterostructure and graphene, showing similar full width at half maximum (FWHM) values of 1.20 ps and 1.45 ps, respectively. With excitation power increasing, the output power grows linearly, and no laser-induced damage is observed at a pump power approaching 92.5 mW (Fig. S4).

### Soliton buildup and evolution in graphene-based SA mode-locked fiber lasers

**Fig. 3:**
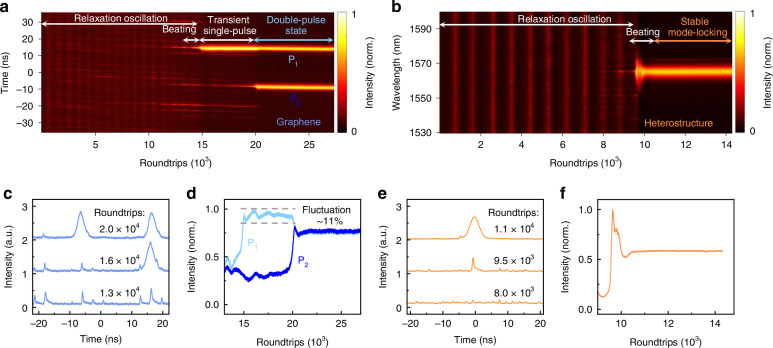
Real-time formation and evolution of solitons in an all-fiber laser mode-locked with the graphene-based SA. Experimental real-time characterization of the entire buildup and evolution processes of double solitons for the graphene-SA (**a**) and of a single soliton for the heterostructure-SA (**b**). There are four evolution stages for the graphene-SA and three stages for the heterostructure-SA, as labeled in the figures. **c** Temporal profiles at representative roundtrips during pulse formation and pulse splitting with the graphene-SA. **d** Retrieved energy of individual pulses obtained with the graphene-SA. Two pulses of the same energy are formed after a violent energy fluctuation. **e** Temporal profiles at representative roundtrips during single-pulse formation with the heterostructure-SA. **f** Retrieved energy of the pulse obtained with the heterostructure-SA. A single-soliton mode-locking state remains stable during propagation (the energy fluctuation is less than 3%)

To further understand the mechanism of mode-locking with the graphene-based SA, we explore the soliton buildup and evolution dynamics via the time-stretch dispersive Fourier transform (TS-DFT) technique^32–34^. Figures 3a, b depict the typical buildup processes of solitons with graphene and the heterostructure as SAs, capturing more than 27,000 and 14,000 consecutive cavity roundtrips, respectively. Both of these SAs can effectively circumvent the Q-switching process prior to mode-locked self-starting^35,36^, thereby preventing potential damage from excessive single-pulse energy exposure. Under identical pumping conditions at 58 mW, the bare graphene-SA and heterostructure-SA exhibit significantly different behaviors. The bare graphene-SA undergoes four distinct stages of evolution: relaxation oscillation, beating dynamics, transient single-pulse formation, and the final double-pulse state (Fig. 3a). Before the first 13,300 roundtrips, multiple pulses with similar intensities energetically compete to form a soliton. Subsequently, background pulses gradually dissipate, and soliton P_1_ emerges, driven by nonlinear pulse shaping and self-phase modulation (Fig. 3c). The transient single-pulse state is quite unstable, with energy fluctuations of approximately 11%, surviving the following ~5000 roundtrips (Fig. 3d). Concurrently, another pulse gains energy from residual background pulses, triggering rapid energy accumulation and leading to the formation of a new soliton P_2_ at ~20,000 roundtrips. The original soliton P_1_ is reshaped and sheds excess energy to P_2_, achieving a more stable state. Ultimately, a double-pulse state forms, with solitons P_1_ and P_2_ exhibiting the same intensity and FWHM, due to the energy quantization effect.

In contrast, the formation of a soliton with the heterostructure-SA proceeds through three stages: relaxation oscillation, beating dynamics, and stable single-soliton mode-locking (Fig. 3b). During the relaxation oscillation stage, background pulses are significantly suppressed, thus avoiding the possibility of multiple soliton generation and promoting stable single-soliton mode-locking. Three selected typical cross-sections, shown in Fig. 3e, emphasize the buildup of pulse formation. A single soliton with clear Kelly sidebands finally forms at nearly 11,000 roundtrips. Its energy remains remarkably steady during propagation, with fluctuations of only approximately 3% (Fig. 3f). No pulse splitting is observed throughout the entire test, indicating a perfect single-soliton mode-locking performance. And the single-shot measurements and averaged spectrum (detected by the spectrometer) profile show remarkable congruence (Fig. S5).

### Robust mode-locking in 2D heterostructure integrated all-fiber lasers

**Fig. 4:**
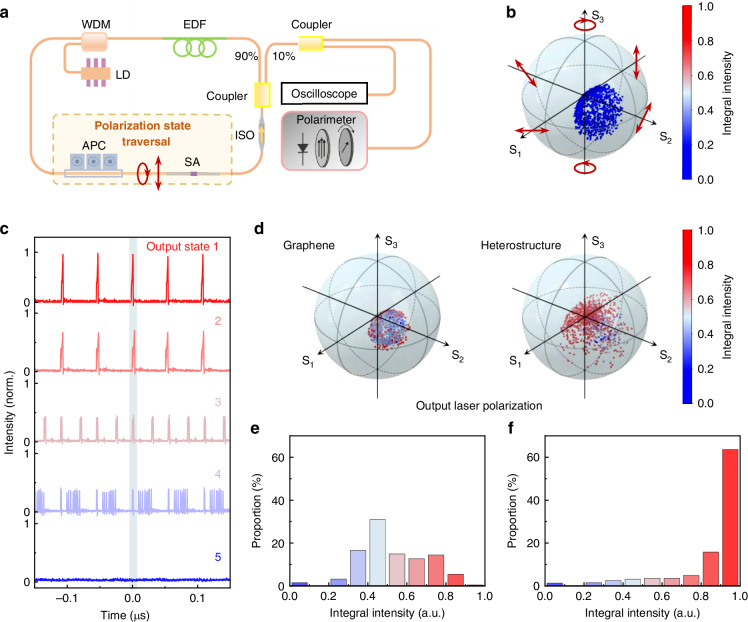
Polarization-dependent mode-locking in the all-fiber laser. **a** Configuration of the mode-locking state measurement system with polarization state traversal. An automatic polarization controller (APC) is used to control the intracavity polarization states. The mode-locking state and output polarization state are measured by an oscilloscope and a polarimeter, respectively. **b** Output polarization state distribution on the Poincaré sphere for the fiber ring cavity without an SA. **c** Typical output laser states on the oscilloscope under different intracavity polarization states. All states are normalized by the maximum intensity of output state 1. The mode-locking state is evaluated by integrating the fundamental peak intensity, which is marked by the blue box around time zero. The integral intensity of state 1 is normalized to 1. The typical output states 1 and 2 (integral intensity > 0.7) are defined as good single-pulse mode-locking. The output states 3, 4, and 5 represent pulse splitting, soliton rain, and a continuous-wave laser, respectively. **d** Output states of the graphene-SA and heterostructure-SA plotted on Poincaré spheres, color-coded by the integral intensity and the degree of polarization as the radius. Statistics on the integral intensities of the graphene-SA (**e**) and heterostructure-SA (**f**). Approximately 20% of the polarization states can maintain single-pulse mode-locking for the graphene-SA, while the value for the heterostructure-SA is approximately 85%

The stable single-soliton buildup dynamics indicate an improvement in the robustness of the heterostructure-SA for mode-locking. To experimentally investigate the polarization tolerance of the bare graphene-SA and heterostructure-SA in mode-locking, we use an automatic polarization controller—equivalent to a continuous rotation of a half-wave plate, quarter-wave plate, and another half-wave plate sequence—to manipulate the intracavity polarization state (Fig. 4a). Polarization states that traverse the entire Poincaré sphere can be obtained (Fig. S6). The output pulse train is monitored by an oscilloscope in real time to evaluate the mode-locking state. Without any SA, no mode-locking operation is achieved (Fig. S7), and the output polarization state distribution is plotted in Fig. 4b. With the bare graphene-SA or heterostructure-SA equipped in the fiber ring cavity, mode-locking operation becomes possible. However, under different intracavity polarization states, various output states are observed, including continuous-wave laser, single-pulse mode-locking, harmonic mode-locking, soliton rain, and pulse splitting (Fig. 4c), which is consistent with previous results^29,30^.

To describe the quality of the output laser states, we integrate the area of the primary pulse at the fundamental repetition rate on the oscilloscope (the area marked around time zero shown in Fig. 4c). Figure 4d shows the Poincaré sphere with spots color-coded by the integral intensity, and the radius value of the spots represents the degree of polarization. The degree of polarization of the output laser is greater, and the integral intensity is generally higher for the heterostructure-SA. Statistics on the integral intensity for the graphene-SA and heterostructure-SA are shown in Fig. 4e, f. Only 20% of the polarization configurations support single-pulse mode-locking for the bare graphene-SA. For the heterostructure-SA, the possibility of pulse splitting is greatly reduced under different polarization states, achieving single-pulse generation in approximately 85% of the configurations.

## Discussion

Typically, mode-locking in a fiber laser is achieved through a combination of saturable absorption and nonlinear polarization rotation effects (Fig. S8)^37,38^. The saturable absorption effect can be quantified by the saturable absorption-related self-amplitude modulation coefficient^3,39^ ${\gamma }_{{SA}}=\frac{{\alpha }_{0}}{{I}_{S}}$. When *γ*_*SA*_ falls below the mode-locking threshold, the nonlinear polarization rotation-related self-amplitude modulation coefficient (*γ*_*NPR*_), which highly depends on polarization and is tunable via a polarization controller, becomes a critical factor influencing the soliton behavior in fiber lasers. This explains why graphene exhibits faint background pulses and an unstable mode-locking output. In the case of the designed heterostructure, *I*_s_ is significantly reduced, leading to an increase in *γ*_*SA*_ from 6.7 × 10^−4^ for bare graphene to 2.3 × 10^−3^, which is a relatively high value in 2D material-based SAs (Table S1). This enhancement ensures that *γ*_*SA*_ exceeds the mode-locking threshold, resulting in high polarization tolerance and even eliminating the necessity for a polarization controller (Fig. S9). High environmental tolerance also guarantees an outstanding long-term stability (Fig. S10), which is essential for practical usage.

In summary, we propose a 2D heterostructure nanocavity integrated on the end facet of an optical fiber to enable robust mode-locking in an ultrafast all-fiber laser. The real-time evolution of the soliton dynamics with the bare graphene-SA and heterostructure-SA is thoroughly analyzed, demonstrating that the nanocavity effectively suppresses background pulses prior to soliton formation, thereby enhancing the mode-locking performance. The 2D heterostructure nanocavity exhibits excellent compatibility with fiber end facet integration, offering promising potential for improving the performance and miniaturization of all-fiber components. By optimizing the heterojunction structure and fiber cavity dispersion^40^, this platform can achieve mode-locking and optical frequency comb generation across multiple bands, making it valuable for communication systems, high-precision sensing, and bio-photonics.

## Materials and methods

### Preparation of 2D materials for optical fiber integration

2D materials, including graphene, MoS_2_, and BN, are mechanically exfoliated from crystals and transferred to a silicon substrate. Van der Waals heterostructures are fabricated by stacking the selected materials in sequence via the standard dry transfer method. Graphene or heterostructures are transferred onto the single-mode fiber (Corning, SMF-28e+) through a standard process by polypropylene carbonate (PPC). The PPC film is peeled off and hangs upside down with the wanted sample at the bottom. After alignment and contact, the sample is released on the fiber core by heating to 130 °C. Residual PPC is removed by acetone. A bare single-mode fiber is placed into a quartz tube and aligned with the 2D material integrated fiber through a homemade alignment system. Covered by refractive index-matched UV-curing resin (Ergo, 8500), the fiber junction is thoroughly cured by a UV lamp. Both ends of the quartz tube are also covered and cured by UV-curing resin for reinforcement. The insertion loss introduced by the encapsulation can be controlled below 10%.

### Characterization of saturable absorption

A pulsed fiber laser (NPI, Rainbow 1550 OEM) is utilized to separately measure the power-dependent transmission of the optical fiber integrated with graphene and heterostructures. The input power is controlled by an electronic variable optical attenuator (Thorlabs, V1550A) positioned after the pulsed fiber laser. Then, the output laser is divided by 90/10 through a fiber output coupler for real-time monitoring of the transmittance (90% for integrated fiber and 10% for bare comparison).

### Implementation and characterization of a mode-locked all-fiber laser

A pump laser (980 nm) is coupled into the fiber ring cavity by a wavelength-division multiplexer (WDM, 980 nm/1550 nm), pumping erbium-doped fiber (60-cm-long, LIEKKI, Er110 4-125). A polarization-independent isolator (ISO) ensures unidirectional operation of the laser in the fiber ring cavity. The graphene and heterostructures are integrated into the cavity separately, serving as a mode-locker to initiate the pulse operation. The pulsed laser is extracted with a ratio of 10% by an output coupler, while the mode-locking pulses are optimized by a manual polarization controller (PC). For a measured output power of 1.1 mW (with pump power of 58 mW), the peak intensity is calculated to be ~2.3 GW/cm^2^ at the graphene layer in the heterostructure-SA, with fiber mode field diameter of ~10 μm, pulse width of ~850 fs, and repetition rate of 13.2 MHz. The time-averaged spectra and temporal pulse profiles are obtained by an optical spectrum analyzer (OSA, Anritsu, MS9740A) and an autocorrelator (APE, PulseCheck USB-15). A radio frequency (RF) spectrum analyzer (Rohde&Schwarz, FSV30) is connected to a photodetector (Electro-Optics Technology Inc., ET-5000F) to measure the RF signals of the output pulse. To implement the TS-DFT technique, the laser is split into two paths by a 70/30 output coupler. One undispersed path records the instantaneous intensity pattern, while the other path stretches the pulse using a dispersion-compensating fiber (DCF, YOFC, ADCM-30) with a total dispersion of −513 ps/nm. Signals from both paths are detected by identical high-speed photodetectors (Discovery Semiconductors, DSC2-30S-39-FC/APC-SMA-2) and captured by a real-time oscilloscope (OSC, SIGLENT, SDS7604AH10) with a sampling rate of 20 GSa/s and a bandwidth of 6 GHz, yielding a resolution of ~0.18 nm for the TS-DFT spectra.

### Characterization of a mode-locked fiber laser in different polarization states

An additional automatic polarization controller (APC, OZ optics, EPC-400) is used to control the intracavity polarization states by changing the voltage of four channels, covering the whole Poincaré sphere. Divided by a 50/50 output coupler, the output laser is introduced into an oscilloscope (OSC, Tektronix, MDO3104) for recording mode-locking states and an infrared polarimeter (Thorlabs, PAX1000IR2/M) for polarization states measurement.

## Supplementary information

Supplementary Information for Robust mode-locking in all-fiber ultrafast laser by nanocavity of two-dimensional heterostructure

## Data Availability

All data are available in the main text or the supplementary materials. Additional data are available from the corresponding authors upon reasonable request.

## References

[1] Liang, C. et al. Ultra stable all-fiber telecom-band entangled photon-pair source for turnkey quantum communication applications. *Opt. Express* **14**, 6936–6941 (2006). 19516877 10.1364/oe.14.006936

[2] Fermann, M. E. & Hartl, I. Ultrafast fibre lasers. *Nat. Photonics* **7**, 868–874 (2013).

[3] Haus, H. A. Mode-locking of lasers. *IEEE J. Sel. Top. Quantum Electron.* **6**, 1173–1185 (2000).

[4] Keller, U. Recent developments in compact ultrafast lasers. *Nature* **424**, 831–838 (2003). 12917697 10.1038/nature01938

[5] Han, Y. et al. Generation, optimization, and application of ultrashort femtosecond pulse in mode-locked fiber lasers. *Prog. Quantum Electron.* **71**, 100264 (2020).

[6] Sarukura, N. et al. cw passive mode locking of a Ti:sapphire laser. *Appl. Phys. Lett.* **56**, 814–815 (1990).

[7] Snitzer, E. & Woodcock, R. 9C8 - saturable absorption of color centers in Nd^3+^ and Nd^3+^ -Yb^3+^ laser glass. *IEEE J. Quantum Electron.* **2**, 627–632 (1966).

[8] Zolotovskaya, S. A. et al. Nd:KGd(WO_4_)_2_ laser at 1.35μm passively Q-switched with V^3+^:YAG crystal and PbS-doped glass. *Opt. Mater.* **28**, 919–924 (2006).

[9] Alcock, A. J. & Walker, A. C. Generation and detection of 150-psec mode-locked pulses from a multi-atmosphere CO_2_ laser. *Appl. Phys. Lett.* **25**, 299–301 (1974).

[10] Haiml, M., Grange, R. & Keller, U. Optical characterization of semiconductor saturable absorbers. *Appl. Phys. B* **79**, 331–339 (2004).

[11] Ortaç, B. et al. Passively mode-locked single-polarization microstructure fiber laser. *Opt. Express* **16**, 2122–2128 (2008). 18542292 10.1364/oe.16.002122

[12] Wang, F. et al. Wideband-tuneable, nanotube mode-locked, fibre laser. *Nat. Nanotechnol.* **3**, 738–742 (2008). 19057594 10.1038/nnano.2008.312

[13] Martinez, A. & Sun, Z. P. Nanotube and graphene saturable absorbers for fibre lasers. *Nat. Photonics* **7**, 842–845 (2013).

[14] Yu, S. L. et al. 2D materials for optical modulation: challenges and opportunities. *Adv. Mater.* **29**, 1606128 (2017). 10.1002/adma.201606128 28220971

[15] Du, J. et al. Phosphorene quantum dot saturable absorbers for ultrafast fiber lasers. *Sci. Rep.* **7**, 42357 (2017). 28211471 10.1038/srep42357 PMC5314455

[16] Bonaccorso, F. et al. Graphene photonics and optoelectronics. *Nat. Photonics* **4**, 611–622 (2010).

[17] Martinez, A. & Yamashita, S. 10 GHz fundamental mode fiber laser using a graphene saturable absorber. *Appl. Phys. Lett.* **101**, 041118 (2012).

[18] Brida, D. et al. Ultrafast collinear scattering and carrier multiplication in graphene. *Nat. Commun.* **4**, 1987 (2013). 23770933 10.1038/ncomms2987

[19] Sotor, J. et al. Sub-90 fs a stretched-pulse mode-locked fiber laser based on a graphene saturable absorber. *Opt. Express* **23**, 27503–27508 (2015). 26480410 10.1364/OE.23.027503

[20] Bao, Q. L. et al. Atomic-layer graphene as a saturable absorber for ultrafast pulsed lasers. *Adv. Funct. Mater.* **19**, 3077–3083 (2009).

[21] Song, Y. W. et al. Graphene mode-lockers for fiber lasers functioned with evanescent field interaction. *Appl. Phys. Lett.* **96**, 051122 (2010).

[22] Sun, Z. P. et al. Graphene mode-locked ultrafast laser. *ACS Nano* **4**, 803–810 (2010). 20099874 10.1021/nn901703e

[23] Chen, J. H. et al. Silica optical fiber integrated with two-dimensional materials: towards opto-electro-mechanical technology. *Light Sci. Appl.* **10**, 78 (2021). 33854031 10.1038/s41377-021-00520-x PMC8046821

[24] Cheng, Y. et al. Controllable growth of graphene photonic crystal fibers with tunable optical nonlinearity. *ACS Photonics* **9**, 961–968 (2022).

[25] Li, X. et al. Saturable absorber based on graphene for a hybrid passive mode-locked erbium-doped fiber laser. *Opt. Fiber Technol.* **70**, 102867 (2022).

[26] Sun, X. L. et al. Tunable ultrafast nonlinear optical properties of graphene/MoS_2_ van der Waals heterostructures and their application in solid-state bulk lasers. *ACS Nano* **12**, 11376–11385 (2018). 30335957 10.1021/acsnano.8b06236

[27] Lee, E. J. et al. Active control of all-fibre graphene devices with electrical gating. *Nat. Commun.* **6**, 6851 (2015). 25897687 10.1038/ncomms7851 PMC4410643

[28] Zaugg, C. A. et al. Ultrafast and widely tuneable vertical-external-cavity surface-emitting laser, mode-locked by a graphene-integrated distributed Bragg reflector. *Opt. Express* **21**, 31548 (2013). 24514728 10.1364/OE.21.031548

[29] Sotor, J. et al. Fundamental and harmonic mode-locking in erbium-doped fiber laser based on graphene saturable absorber. *Opt. Commun.* **285**, 3174–3178 (2012).

[30] Han, M. M. et al. Polarization dynamic patterns of vector solitons in a graphene mode-locked fiber laser. *Opt. Express* **23**, 2424–2435 (2015). 25836110 10.1364/OE.23.002424

[31] Grudinin, A. B. & Gray, S. Passive harmonic mode locking in soliton fiber lasers. *J. Opt. Soc. Am. B* **14**, 144–154 (1997).

[32] Herink, G. et al. Real-time spectral interferometry probes the internal dynamics of femtosecond soliton molecules. *Science* **356**, 50–54 (2017). 28386005 10.1126/science.aal5326

[33] Liu, X. M., Yao, X. K. & Cui, Y. D. Real-time observation of the buildup of soliton molecules. *Phys. Rev. Lett.* **121**, 023905 (2018). 30085749 10.1103/PhysRevLett.121.023905

[34] Liu, X. M. & Cui, Y. D. Revealing the behavior of soliton buildup in a mode-locked laser. *Adv. Photonics* **1**, 016003 (2019).

[35] Ryczkowski, P. et al. Real-time full-field characterization of transient dissipative soliton dynamics in a mode-locked laser. *Nat. Photonics* **12**, 221–227 (2018).

[36] Liu, X. M., Popa, D. & Akhmediev, N. Revealing the transition dynamics from *Q* switching to mode locking in a soliton laser. *Phys. Rev. Lett.* **123**, 093901 (2019). 31524444 10.1103/PhysRevLett.123.093901

[37] Ippen, E. P. Principles of passive mode locking. *Appl. Phys. B* **58**, 159–170 (1994).

[38] Chen, T. H. et al. Incorporating MoS_2_ saturable absorption with nonlinear polarization rotation for stabilized mode-locking fibre lasers. *Laser Phys. Lett.* **15**, 075102 (2018).

[39] Kurtner, F. X., Der Au, J. A. & Keller, U. Mode-locking with slow and fast saturable absorbers-what’s the difference?. *IEEE J. Sel. Top. Quantum Electron.* **4**, 159–168 (1998).

[40] Zhang, H. et al. Compact graphene mode-locked wavelength-tunable erbium-doped fiber lasers: from all anomalous dispersion to all normal dispersion. *Laser Phys. Lett.* **7**, 591–596 (2010).

