# Supplementary material for: Robust mode-locking in all-fiber ultrafast laser by nanocavity of two-dimensional heterostructure
Source: Light Sci Appl. 2025 Sep 3;14:301. doi: 10.1038/s41377-025-02018-2 (PMC12408808; doi:10.1038/s41377-025-02018-2)
Supplement: Supplementary file 1 — Supplementary Information for Robust mode-locking in all-fiber ultrafast laser by nanocavity of two-dimensional heterostructure [file 41377_2025_2018_MOESM1_ESM.pdf]

Supplementary Information for

Robust mode-locking in all-fibre ultrafast laser by nanocavity of two-dimensional heterostructure

Jiahui Shao, Guangjie Yao, Xuecheng Wu, Kaifeng Lin, Shaoyi Zhang, Xu Cheng, Ding Zhong, Chang Liu, Can Liu, Fengqiu Wang*, Kaihui Liu* and Hao Hong*

*Corresponding author: Hao Hong, haohong@pku.edu.cn;
Kaihui Liu, khliu@pku.edu.cn;
Fengqiu Wang, fwang@nju.edu.cn

This PDF file includes:

Supplementary Figs. 1-10

Supplementary Table 1

References

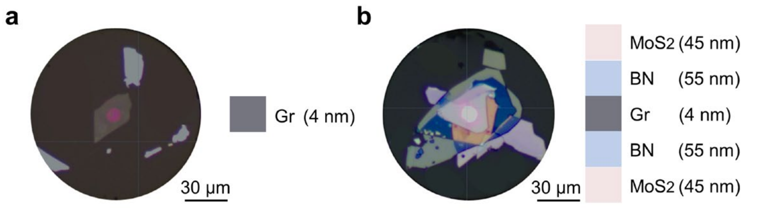

Fig. S1 Optical images of 2D materials integrated on optical fibre. a Optical image of bare graphene integrated on optical fibre. **b** Optical image of heterostructure (with an BN thickness of 55 nm) integrated on optical fibre.

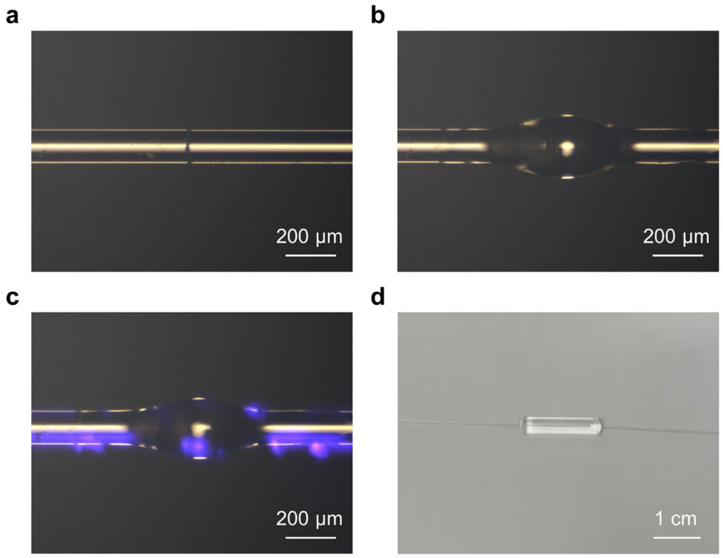

Fig. S2 Schematics for the encapsulation of optical fibre integrated with 2D materials. a Optical fibres are aligned using multi-axis stages with power monitored to achieve maximum transmittance. **b** The fibre junction is covered with UV curing resin. **c** The UV curing resin is cured by a UV light. **d** The fibre junction is encapsulated inside a quartz tube for enhancement.

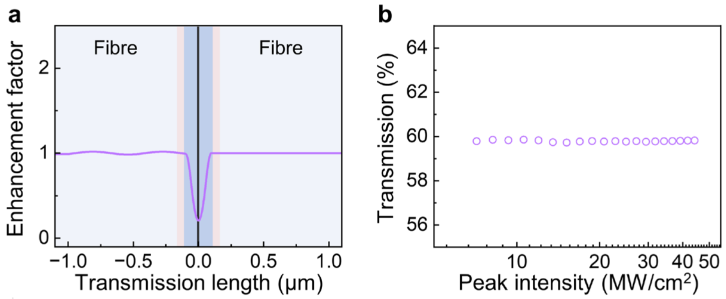

Fig. S3 Internal optical field intensity distribution (**a**) **and transmission measurement** (**b**) **of the heterostructure (with an BN thickness of 55 nm). a** The optical field intensity at graphene position is significantly suppressed. **b** No obvious saturable absorption effect is observed.

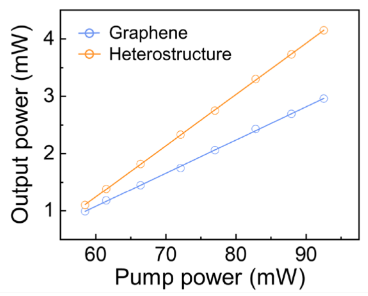

Fig. S4 Fibre laser output power as a function of pump power with the graphene-SA and heterostructure-SA integration. With excitation power increasing, the output power grows linearly. No photodamage is observed when the pump power (980 nm) reaches 92.5 mW.

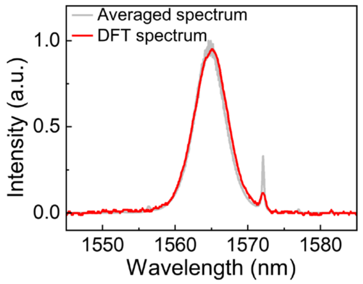

Fig. S5 The comparison of averaged spectrum (grey line) and DFT spectrum (red line) with heterostructure-SA. The observed relative attenuation of Kelly sidebands in DFT trace originates from fundamental detection constraints of continuous-wave components inherent to DFT approach.

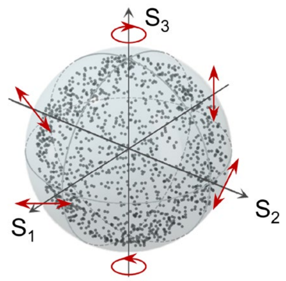

Fig. S6 Output polarization states distribution on the Poincaré sphere with the modulation of automatic polarization controller (APC). Nearly full polarization states can be achieved in our experiments.

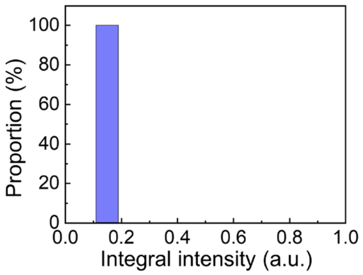

Fig. S7 Statistics on integral intensity of the fibre laser output states without any SA. When intracavity polarization states are traversed with APC, no mode-locking state is achieved by nonlinear polarization rotation effect merely in the fibre laser without SA.

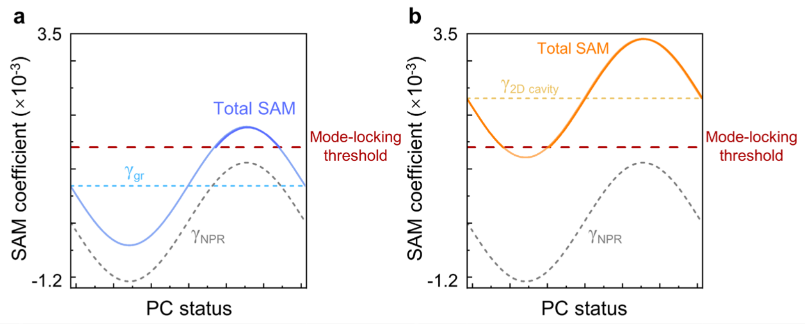

Fig. S8 Schematics for the self-amplitude modulation (SAM) coefficient combined of nonlinear polarization rotation (NPR) from polarization controller (PC) and saturable absorption. a When bare graphene-SA is incorporated, the intracavity total SAM coefficient (blue solid line) is contributed by NPR (grey dashed line) and weak saturable absorption (blue dashed line). Variations in the PC status lead to changes in total SAM coefficient due to NPR. Mode-locking is achieved once the total SAM coefficient exceeds the mode-locking threshold (red dashed line). **b** When heterostructure-SA is incorporated, the high SAM coefficient for saturable absorption causes higher intracavity total SAM coefficient (orange solid line), thus promoting mode-locking operation under most PC status.

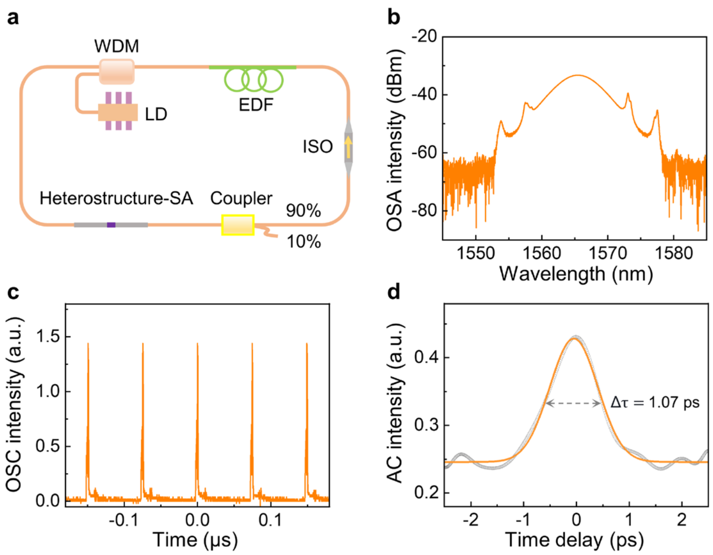

Fig. S9 Ultrafast all-fibre laser based on heterostructure-SA without polarization controller. **a** Schematic of the mode-locked all-fibre laser. The optical components consist of a laser diode (LD), wavelength-division multiplexer (WDM), erbium-doped fibre (EDF), isolator (ISO) and heterostructure-SA. **b** Spectrum of the output laser. The central wavelength and spectral bandwidth (Δλ) are 1565.5 nm and 5.7 nm, respectively. **c** Output pulse train from the all-fibre laser. The repetition rate is 13.4 MHz. **d** Autocorrelation trace with full width at half maximum of ~1.07 ps, fitted by a Gaussian function.

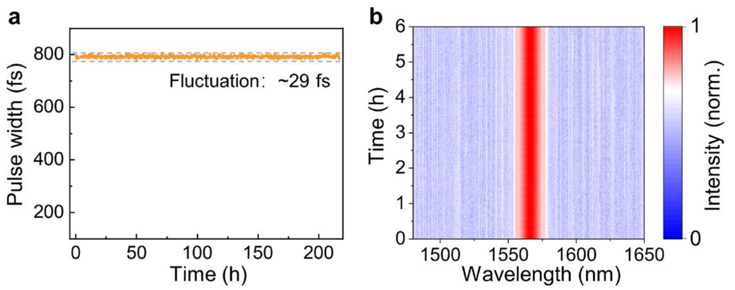

Fig. S10 Long-term stability of fibre laser mode-locked by heterostructure-SA. a Pulse width of the fibre laser remained stable under continues operation. Data points were collected every 15 minutes over the entire 9-day test period, with a total fluctuation of less than 30 fs. **b** Spectral stability of the ultrafast fibre laser. Data points were collected every 20 seconds during the 6-hour test period, with no spectral shift observed.

Table S1 | Comparison of 2D material-based saturable absorbers

Material	Wavelength (nm)	α_0 (%)	I_s (MW cm^{-2})	γ_{SA} ($\times 10^{-4}$)	Reference
**Graphene**	1566	4.2	62.9	6.7	This work
**Heterostructure**	**1565**	**5.0**	**22.0**	**22.7**	**This work**
**Graphite**	1567	3.3	53	6.2	Ref.[1]
**Graphene**	1559	1.3	266	0.5	Ref.[2]
**Graphene**	1608	0.2	75.6	0.3	Ref.[3]
**Graphene**	1570	5.7	61.9	9.2	Ref.[4]
**Graphene**	1559	2.9	53.2	5.4	Ref.[5]
**MoS_2**	1556	2.7	137	2.0	Ref.[6]
**MoS_2**	1570	4.3	34	12.6	Ref.[7]
**WS_2**	1557	1.0	600	0.2	Ref.[8]
**WS_2**	1558	1.2	25	4.8	Ref.[9]
**BP**	1555	10.0	15.0	66.7	Ref.[10]
**GaSe**	1555	6.4	72	8.9	Ref.[11]
**Sb_2Te_3**	1565	6	31	19.3	Ref.[12]

References

1. Steinberg, D. et al. Mechanically exfoliated graphite onto D-shaped optical fiber for femtosecond mode-locked erbium-doped fiber laser. *Journal of Lightwave Technology* **36**, 1868–1874 (2018).
2. Sun, Z. P. et al. Graphene mode-locked ultrafast laser. *ACS Nano* **4**, 803–810 (2010).
3. Park, N. H. et al. Monolayer graphene saturable absorbers with strongly enhanced evanescent-field interaction for ultrafast fiber laser mode-locking. *Optics Express* **23**, 19806–19812 (2015).
4. Chen, T.-H. et al. Unintentional polarization dependent pulsewidth of graphene mode-locked Er-Doped fiber lasers. *IEEE Journal of Selected Topics in Quantum Electronics* **23**, 50–59 (2017).
5. Huang, P. L. et al. Stable mode-locked fiber laser based on CVD fabricated graphene saturable absorber. *Optics Express* **20**, 2460–2465 (2012).
6. Wu, K. et al. 463-MHz fundamental mode-locked fiber laser based on few-layer MoS_2 saturable absorber. *Optics Letters* **40**, 1374–1377 (2015).
7. Liu, H. et al. Femtosecond pulse erbium-doped fiber laser by a few-layer MoS_2 saturable absorber. *Optics Letters* **39**, 4591–4594 (2014).
8. Mao, D. et al. WS_2 mode-locked ultrafast fiber laser. *Scientific Reports* **5**, 7965 (2015).
9. Yan, P. G. et al. Microfiber-based WS_2-film saturable absorber for ultra-fast photonics. *Optical Materials Express* **5**, 479–489 (2015).
10. Jin, X. X. et al. 102 fs pulse generation from a long-term stable, inkjet-printed black phosphorus-mode-locked fiber laser. *Optics Express* **26**, 12506–12513 (2018).
11. Li, Y. et al. GaSe saturable absorber for mode-locked Er-doped fiber laser. *Infrared Physics & Technology* **96**, 325–329 (2019).
12. Sotor, J., Sobon, G. & Abramski, K. M. Sub-130 fs mode-locked Er-doped fiber laser based on topological insulator. *Optics Express* **22**, 13244–13249 (2014).
